# On solving mutual liability problems

**DOI:** 10.1007/s00186-017-0621-1

**Published:** 2017-11-07

**Authors:** Mirjam Groote Schaarsberg, Hans Reijnierse, Peter Borm

**Affiliations:** 1 TNO, Netherlands Organisation for Applied Scientific Research, The Hague, The Netherlands; 2 Center and Department of Econometrics and Operations Research, Tilburg University, Tilburg, The Netherlands

**Keywords:** Mutual liability problems, Bankruptcy, Monetary interrelationships in financial crises, C71, G33

## Abstract

This paper introduces mutual liability problems, as a generalization of bankruptcy problems, where every agent not only owns a certain amount of cash money, but also has outstanding claims and debts towards the other agents. Assuming that the agents want to cash their claims, we will analyze mutual liability rules which prescribe how the total available amount of cash should be allocated among the agents. We in particular focus on bilateral $\varphi$-transfer schemes, which are based on a bankruptcy rule $\varphi$. Although in general a $\varphi$-transfer scheme need not be unique, we show that the resulting $\varphi$-transfer allocation is. This leads to the definition of $\varphi$-based mutual liability rules. For so called hierarchical mutual liability problems an alternative characterization of $\varphi$-based mutual liability rules is provided. Moreover it is shown that the axiomatic characterization of the Talmud rule on the basis of consistency can be extended to the corresponding mutual liability rule.

## Introduction

The classical bankruptcy problem, consisting of a single estate and multiple claimants, is introduced by O’Neill (1982). A bankruptcy rule prescribes, for each bankruptcy problem, how to divide the estate over the claimants. In the literature one can find a wide variety of bankruptcy rules, which arise from both an axiomatic as well as a game-theoretic analysis, see for an overview, Thomson (2003, 2015). The classical bankruptcy problem has been extended in various ways. Without trying to provide a complete overview, we mention extensions to multi-issue allocation situations in which the estate has to be divided among a group of agents with claims stemming from different issues, see Calleja et al. (2005), to stochastic bankruptcy games (Habis and Herings 2013), to rationing problems in the presence of baselines (see Hougaard et al. 2013a, b), and to allocating the losses due to financial distress within a business sector (Van Gulick 2010). Lately, a main trending topic is multiple estates. In a current work of Bjorndal and Jornsten (2010) a bankruptcy problem with multiple banks (estates) is represented by a flow model. The banks can have separate claims on each other and there is a set of agents having separate claims on those banks. Pálvölgyi et al. (2014) consider the case of agents with non-homogeneous preferences over multiple estates. Here, the agents have a single claim, but the utility per estate differs. The problem is analyzed from a non-cooperative perspective and focusses on how the agents should divide their claim into subclaims over the estates. Moulin and Sethuraman (2013) analyze bipartite rationing problems with multiple estates, agents with a single claim, but in which the agents are not necessarily compatible with all estates. These compatibilities are represented by a bipartite graph. By analyzing the flows in the graph and using a consistency axiom, bankruptcy rules are extended to this setting.

In this paper we introduce mutual liability problems with multiple estates of a rather different nature. In financial accounting a *liability* is defined as an obligation of “an entity arising from past transactions or events, the settlement of which may result in the transfer or use of assets, provision of services or other yielding of economic benefits in the future.”^1^ Usually a liability is associated with an uncertainty, but this need not be the case. The more creditors an agent has, the higher the liabilities. We will investigate the scenario where a group of agents is related by having mutual liabilities, but reaches the point in time where the agents want to cash their claims. Before this time moment, none of the agents worry about the possible insufficient cash in the current assets. Until, for some exogenous reason, individuals start cashing their claims. This will lead to a cascading effect and will reveal the possibly insufficient cash level of agents and the agents typically might not obtain all of what they, however rightfully, claim.

This approach can be applied to financial networks and can be seen as a simplified and deterministic model of the monetary interrelationships between banks, governments and companies in case of a financial crisis and threatening bankruptcy of banks. We refer to Eisenberg and Noe (2001) for an overview, and to Glasserman and Young (2015) and Acemoglu et al. (2015) for further issues on (in)stability and contagion in financial networks. Mutual liabilities also relate to so-called claims problems with circular priorities as mentioned in e.g. Benson (1935) and Kocourek (1935). These papers consider the legal perspective on interdependent claims, whereas we consider the mathematical perspective using the current knowledge of bankruptcy problems. Mutual liabilities in a discrete and decentralized financial network setting are analyzed in Csóka and Herings (2016, 2017), whereas we consider a continuous and centralized framework.

A *mutual liability problem* can be represented by a matrix, in which an entry represents a claim from one agent on another agent. The diagonal entries represents the players’ cash levels. A special class of mutual liability problems are the *hierarchical mutual liability problems* in which the claim matrix is triangular. This implies that we can index the agents, such that every agent only claims from agents with lower index. In this sense there is a hierarchy among the agents. For an example, think of the vertical relations in a supply-chain: insufficient cash of a buyer may lead to insufficient cash of his supplier(s).

This paper will analyze mutual liability problems from an allocation perspective: if in a mutual liability problem the agents reach the stage that they want to cash their claims and remove all current liabilities, how should the total amount of available cash be fairly distributed among the agents? At this moment we refrain from a formal cooperative game theoretic approach. In our opinion one should first analyze the numerous new intricacies of our new model on the level of individual agents before attempting a coalitional approach. Instead, we implicitly assume that there is an independent authority charged with the task of fairly solving the mutual liability problem. A *mutual liability rule* will for each mutual liability problem prescribe how to allocate the total cash among the agents. Since the choice of the independent authority for such a rule might well be inspired by underlying bilateral transfers (as are the claims)—and, in practice, will be quite hard to defend without them,—we assume each allocation to stem from a so-called *(bilateral) transfer scheme*. In fact we show that, under a weak condition (called reasonability), every allocation can be supported be such a transfer scheme. More specifically, given a bankruptcy rule $\varphi$, we consider $\varphi$-*transfer schemes*, in which the incoming plus available cash of every agent is allocated among his claimants according to the bankruptcy rule $\varphi$. We show that for every bankruptcy rule $\varphi$ there always exists a $\varphi$-transfer scheme, which is not necessarily unique. Interestingly, it is seen that each $\varphi$-transfer scheme leads to the same $\varphi$-*transfer allocation*, so allocation-wise a unique outcome is provided. For the subclass of hierarchical mutual liability problems, it is seen that the $\varphi$-transfer scheme is also unique.

These results imply that each bankruptcy rule $\varphi$ can be extended to a mutual liability rule: a $\varphi$-*based mutual liability rule*. We provide an explicit characterization for the $\tau$-based mutual liability rule based on the Talmud rule $\tau$ (Aumann and Maschler 1985), by extending the properties of consistency and the concede and divide-principle from the bankruptcy setting to the context of mutual liability problems.

Profiting from the special structure of hierarchical mutual liability problems, one can extend bankruptcy rules in an alternative recursive way into mutual liability rules. It is shown that for each bankruptcy rule $\varphi$, the resulting allocation coincides with the unique transfer allocation prescribed by the corresponding $\varphi$-based mutual liability rule, thus providing another characterization of $\varphi$-based mutual liability rules on the class of hierarchical mutual liability problems.

The paper concludes with a brief discussion of the assumptions underlying a mutual liability problem and sketches an alternative approach to solve mutual liability problems, which involves reducing non-hierarchical problems into more tractable hierarchical mutual liability problems by bilaterally and cyclically leveling the claims. We will see, however, that there is no straightforward procedure how to eliminate the cycles and that different procedures may result in different reduced problems.

The organization of this paper is as follows. In Sect. 2 we will formally introduce mutual liability problems. Then, in Sect. 3 we will give a short introduction to bankruptcy rules $\varphi$, define transfer schemes, $\varphi$-transfer schemes and corresponding transfer allocations. Section 4 studies mutual liability rules and in particular $\varphi$-based mutual liability rules in a hierarchical setting, while Sect. 5 analyzes $\varphi$-based mutual liability rules on the general class of mutual liability problems, including the characterization of the $\tau$-based mutual liability rule. Section 6 concludes.

## Mutual liability problems and mutual liability rules

A classical bankruptcy problem involves an estate *E* that has to be divided among a finite group of agents *N*, all having a nonnegative claim $d_i, i\in N$, on the estate. We summarize these claims into a vector $d=(d_i)_{i\in N}$. The set of all bankruptcy problems (*E*, *d*) on *N* is denoted by ${\mathcal {B}}^N$. For expositional reasons, we do not impose that $\sum _{i\in N}d_i>E$ for a bankruptcy problem $(E,d)\in {\mathcal {B}}^N$. Of course, if $\sum _{i\in N}d_i\le E$, then any bankruptcy rule will lead to the allocation $d \in {\mathbb {R}}^N$.

In a mutual liability problem, a finite group of economic agents, denoted by *N*, have been interacting for a certain time period. Their past economic transactions have resulted in a situation in which the agents have nonnegative claims on each other (think of debtors and creditors or accounts payable and receivable). As in bankruptcy problems, we assume that these claims are known, rightful and justifiable. Further, every agent has a certain nonnegative cash level or cash reserve with which he can (partially) pay his possible debtors. A *mutual liability problem* can be represented by a nonnegative matrix $C\in {\mathbb {R}}_{+}^{N \times N}$. Here each cell $c_{ij}\in C$ represents the claim of agent *j* on agent *i*, $i\ne j$, and $c_{ii}$ represents the cash level of agent *i*. If

$$\begin{aligned} \sum _{i\in N}c_{ii}< \sum _{i,j\in N, i\ne j}c_{ij}, \end{aligned}$$

there is not sufficient cash to fulfill all the claims. If for some agent $i\in N$,

$$\begin{aligned} \sum _{j\in N}c_{ji}-\sum _{j\in N\backslash \{i\}} c_{ij}<0, \end{aligned}$$

agent *i* will never be able to satisfy all his claimants. We will, however, not impose any restrictions except nonnegativity on the matrix *C* beforehand. The main question is how to divide $\sum _{i\in N} c_{ii}$ over the agents in *N*.

We denote by ${\mathcal {L}}^N$ the set of all mutual liability problems on *N*. A *mutual liability rule* (LR) $f:{\mathcal {L}}^N\rightarrow {\mathbb {R}}^{N}$ is such that $f(C)\ge 0$ and $\sum _{i\in N} f_i(C)=\sum _{i\in N} c_{ii}$ for all $C\in {\mathcal {L}}^N$.

We will distinguish a class of mutual liability problems with a special triangular structure. A mutual liability problem $C\in {\mathcal {L}}^N$ is called a *hierarchical mutual liability problem* if, by reordering the agents, *C* can be transformed into an upper triangular matrix with zeros below the diagonal. The set ${\mathcal {L}}^{N,\Delta }$ contains all hierarchical mutual liability problems on *N*. A mutual liability rule that is defined on the domain of hierarchical mutual liability problems is called a *hierarchical mutual liability rule*.

### Example 2.1

Let $N=\{1,2,3\}$ and $C\in {\mathcal {L}}^N$ be given byThe matrix should be interpreted in the following way. Agent 1 has a cash level of 3. He has a claim of 2 on agent 2 and a claim of 1 on agent 3, while agent 2 and 3 have a claim of 1 and 4 on agent 1. Agent 2 has a cash level of 2. He has no further claims, than the 1 on agent 1 we already mentioned, but agent 1 and 3 have a combined claim of 8 on him. This means in particular that agent 2 will never be able to pay off his debts. Agent 3 has a cash level of 1, agent 1 has a claim of 1 on his cash, while agent 3 has a claim of 4 on agent 1 and a claim of 6 on agent 2. $\square$

### Example 2.2

Let $N=\{1,2,3,4\}$ and $C\in {\mathcal {L}}^N$ be given by

$$\begin{aligned} C=\begin{bmatrix} 4&2&4&4\\0&3&0&1\\ 0&0&2&3\\ 0&0&0&2\end{bmatrix}. \end{aligned}$$

The claim matrix is upper triangular, since agent 1 only faces claims and has no claims on agents 2, 3 or 4. Furthermore, agent 2 has a claim on agent 1 but faces claims only from agents 3 and 4. Agent 3 has a claim on agent 1 and faces a claim of only agent 4, while agent 4 faces no claims at all, but he has a claim on all other three agents. $\square$

Mutual liability problems can be seen as a generalization of bankruptcy problems. Each bankruptcy problem $(E,d)\in {\mathcal {B}}^N$ with $N=\{1,2,\ldots ,n\}$ corresponds to a hierarchical mutual liability problem $C(E,d)\in {\mathcal {L}}^{\bar{N}}$ with $\bar{N}=N\cup \{0\}$ given by


## Transfer schemes and $\varphi$-transfer schemes

Before elaborating on transfer schemes, we provide some details on bankruptcy rules and the Talmud rule in particular.

### Bankruptcy rules $\varphi$ and the Talmud rule $\tau$

A *bankruptcy rule* $\varphi :{\mathcal {B}}^N\rightarrow {\mathbb {R}}^N$ assigns to every bankruptcy problem $(E,d)\in {\mathcal {B}}^N$ a vector $\varphi (E,d)\in {\mathbb {R}}^N$, such that

1 $$\begin{aligned} \sum _{i\in N}\varphi _i(E,d)=\min \left\{ E,\sum _{j\in N}d_j\right\} , \end{aligned}$$

$0\le \varphi (E,d)\le d$ and such that *estate monotonicity* is satisfied: for all $(E,d)\in {\mathcal {B}}^N$ and all $(E',d)\in {\mathcal {B}}^N$ with $E'\ge E$, we have $\varphi (E,d)\le \varphi (E',d)$. Note that the class ${\mathcal {B}}^N$ also contains bankruptcy problems (*E*, *d*) in which *E* is sufficient to fulfill the claims *d*. For such problems, $\varphi (E,d)=d$ for any bankruptcy rule $\varphi$. Please note that we require estate monotonicity from the outset. The advantage of this assumption is that any bankruptcy rule is *continuous* in the estate: for a sequence of nonnegative estates $E_1,E_2,\ldots$ that converges to *E* and for any nonnegative claim vector $d\in {\mathbb {R}}^N$, the sequence $\varphi (E_1,d),\varphi (E_2,d),\ldots$ converges to $\varphi (E,d)$.

For a detailed overview on bankruptcy rules we refer to Thomson (2003, 2015). Our focus will be mainly on the Talmud rule $\tau$ (Aumann and Maschler 1985), which is based on the Constrained Equal Awards rule.

The Constrained Equal Awards rule *CEA* is, for all $(E,d)\in {\mathcal {B}}^N$ and all $i\in N$, defined by

$$\begin{aligned} CEA_i(E,d)=\min \{\lambda ,d_i\}, \end{aligned}$$

where $\lambda \in {\mathbb {R}}$ is such that $\sum _{i\in N}\min \{\lambda ,d_i\}=\min \{E,\sum _{j\in N}d_j\}$.

The Talmud rule $\tau$ is, for all $(E,d)\in {\mathcal {B}}^N$, defined by

$$\begin{aligned} \tau (E,d)={\left\{ \begin{array}{ll} d &{} \text { if } \sum \limits _{j\in N} d_j\le E,\\ d-CEA\left( \sum \limits _{j\in N}d_j-E,\frac{1}{2}d\right) &{} \text { if } E<\sum \limits _{j\in N} d_j < 2E,\\ CEA\left( E,\frac{1}{2}d\right) &{} \text { if } \sum \limits _{j\in N}d_j\ge 2E. \end{array}\right. } \end{aligned}$$

For bankruptcy problems involving two agents, $\tau$ satisfies the concede and divide-principle $c \& d$ [cf. Thomson (2003)]. This means that for $(E,d)\in {\mathcal {B}}^N$ with $N=\{1,2\}$,

$$\begin{aligned} \tau _1(E,d)={\left\{ \begin{array}{ll} (E-d_2)^+ +\frac{E-(E-d_1)^+ - (E-d_2)^+}{2} &{}\text { if } d_1+d_2 \ge E,\\ d_1 &{}\text { if } d_1+d_2<E, \end{array}\right. } \end{aligned}$$

where $(x)^+=\max \{x,0\}$ for all $x\in {\mathbb {R}}$. Here $(E-d_2)^+$ represents the part of the estate conceded to agent 1 by agent 2, while $\frac{E-(E-d_1)^+ - (E-d_2)^+}{2}$ indicates that the total amount of the estate that is not conceded, is divided equally.

So far, bankruptcy rules are defined on a fixed but arbitrary finite agent set *N*. Alternatively, bankruptcy rules can also be viewed as rules on the class ${\mathcal {B}}$ of bankruptcy problems with arbitrary but finite *N*. On the class ${\mathcal {B}}$, $\tau$ can be characterized by means of the $c \& d$-principle and the property of consistency.

Here, a bankruptcy rule $\varphi$ on ${\mathcal {B}}$ is called *consistent* if for each finite agent set *N*, each $(E,d)\in {\mathcal {B}}^N$ and all $T\in 2^N\backslash \{\emptyset \}$, we have

$$\begin{aligned} \varphi (E,d)_{|_T}=\varphi \left( \sum _{j\in T}\varphi _j(E,d),d_{|_T}\right) . \end{aligned}$$

Here, the subscript $_{|_T}$ to a vector *x* refers to its projection to the subspace ${\mathbb {R}}^T: x_{|_T}=y\in {\mathbb {R}}^T$ for that *y* with $y_i=x_i$ for all $i\in T$. Note that $(\sum _{j\in T}\varphi _j(E,d),d_{|_T})\in {\mathcal {B}}^T$.

Consistency of a rule requires that a possible reallocation of the total amount which has been allocated to a coalition *T*, on the basis of to the same bankruptcy rule, does not change the initial individual allocations within this coalition.

### Towards transfer schemes

To devise mutual liability rules, we will explicitly consider (bilateral monetary) transfer schemes on which the allocations prescribed by the rule are based.

#### Definition A

Let $C\in {\mathcal {L}}^N$. Then, the matrix $P=(p_{ij})\in {\mathbb {R}}^{N\times N}$ is a *transfer scheme* for *C*, if

(i) for all $$i\in N$$, $$p_{ii}=c_{ii}$$,
(ii) for all $i,j\in N$ with $i\ne j$, $0\le p_{ij}\le c_{ij}$,
(iii) for all $i\in N$, $\sum _{j\in N\backslash \{i\}}p_{ij}\le p_{ii}+\sum _{j\in N\backslash \{i\}}p_{ji}$.

Let ${\mathcal {P}}(C)$ denote the set of all possible transfer schemes for *C*.

The interpretation of a transfer scheme $P=(p_{ij})\in {\mathbb {R}}^{N\times N}$ for the mutual liability problem $C\in {\mathcal {L}}^N$ is the following: $p_{ij}$, $i\ne j$, corresponds to the monetary transfer from agent *i* to *j*. For technical reasons and for computational convenience we require (i). Condition (ii) states that the payment $p_{ij}$ is nonnegative, but not higher than claim $c_{ij}$ of agent *j* on *i*. Condition (iii) requires that the sum of outgoing payments of *i* does not exceed his available cash plus incoming payments.

A transfer scheme directly leads to an allocation of the available cash.

#### Definition B

Let $C\in {\mathcal {L}}^N$ and let $P\in {\mathcal {P}}(C)$. Then $\alpha ^P\in {\mathbb {R}}^N$ is called the *transfer allocation based on P* if, for all $i\in N$,

$$\begin{aligned} \alpha ^P_i=p_{ii}+\sum _{j\in N\backslash \{i\}}(p_{ji}-p_{ij}). \end{aligned}$$

Note that transfer allocations $\alpha ^{P}$ are nonnegative because of (iii) in Definition A and efficient due to the fact that

$$\begin{aligned} \sum _{i\in N}\alpha ^P_i=&\sum _{i\in N}\big [p_{ii}+\sum _{j\in N\backslash \{i\}}(p_{ji}-p_{ij})\big ]\\ =&\sum _{i\in N}p_{ii}+\sum _{i\in N}\sum _{j\in N\backslash \{i\}}p_{ji}-\sum _{i\in N}\sum _{j\in N\backslash \{i\}}p_{ij}\\ =&\sum _{i\in N}p_{ii}=\sum _{i\in N}c_{ii}. \end{aligned}$$

#### Example 3.1

Reconsider the mutual liability problem $C\in {\mathcal {L}}^N$ of Example 2.1 with $N=\{1,2,3\}$ and *C* given by

$$\begin{aligned} C=\begin{bmatrix} 3&\quad 1&\quad 4 \\ 2&\quad 2&\quad 6 \\ 1&\quad 0&\quad 1 \end{bmatrix}. \end{aligned}$$

An example of a transfer scheme for *C* is

$$\begin{aligned} P=\begin{bmatrix} 3&\quad 1&\quad 4 \\ 1.5&\quad 2&\quad 1.5 \\ 1&\quad 0&\quad 1 \end{bmatrix}. \end{aligned}$$

Conditions (i) and (ii) of Definition A can easily be checked. To verify condition (iii) of Definition A, observe that

$$\begin{aligned} p_{12}+p_{13}&=5\le p_{11}+p_{21}+p_{31}=5.5\\ p_{21}+p_{23}&=3\le p_{22}+p_{12}+p_{32}=3\\ p_{31}+p_{32}&=1\le p_{33}+p_{13}+p_{23}=6.5. \end{aligned}$$

Note that *P* leads to the allocation $\alpha ^P=(0.5,0,5.5)$. $\square$

Let $C\in {\mathcal {L}} ^N$ represent a mutual liability problem. An allocation *x* is said to be *reasonable from below* (with respect to *C*) if for all $S\subset N$

2 $$\begin{aligned} x(S)\ge \sum _{i\in S} \big ( c_{ii}-\sum _{j\in N{\setminus } S} c_{ij} \big ). \end{aligned}$$

This is a core type of inequality in the sense that it sounds reasonable that if coalition *S* receives even less than the right hand side of (2), it better could forfeit the obligations from $N{\setminus } S$ that members of *S* possess, pay its liabilities to $N{\setminus } S$, and settle their interior liabilities on their own.

An allocation *x* is said to be *reasonable from above* if for all $T\subset N$

3 $$\begin{aligned} x(T)\le \sum _{j\in T} \left( c_{jj}+\sum _{i\in N{\setminus } T} c_{ij} \right) . \end{aligned}$$

An allocation is reasonable from below if and only if it is reasonable from above. In order to show this, substitute $S=N{\setminus } T$ in (2). Hence, we simply call an allocation *reasonable* if it is reasonable from below.

A mutual liability rule *f* on ${\mathcal {L}}^N$ is called *reasonable* if for all $C \in {\mathcal {L}}^N$, all $x\in f (C)$ are reasonable. Since reasonability is a desirable property, the theorem below shows that it is not restrictive to only consider allocations that arise from transfer schemes.

#### Theorem 3.2

Let $C\in {\mathcal {L}} ^N$ be a mutual liability problem. Let *x* be an allocation. Then there exists a transfer scheme $P\in {\mathcal {P}} (C)$ with $\alpha ^P(C)=x$ if and only if *x* is reasonable.

#### Proof

The ‘only if’-part is a direct consequence of condition (iii) in Definition A. In order to prove the ‘if’-part, the well known Max-flow-min-cut theorem is applied. Construct a directed network as follows. The set of vertices *V* consists of a source $\text {So}$, a sink $\text {Si}$, and one node for each agent: $V=\{\text {So},\text {Si}\}\cup N$. The arc set *A* is the complete digraph on *V*, without loops. Each arc has a capacity, given as follows:

$$\begin{aligned} \begin{array}{lll} \text {cap}(\text {So},\text {Si}) &{}= 0, &{}\\ \text {cap}(\text {So},i) &{}= c_{ii} &{}\text {for all } i\in N, \\ \text {cap}(i,j) &{} = c_{ij} &{}\text {for all } i,j\in N, i\ne j,\\ \text {cap}(j,\text {Si}) &{}= x_j &{}\text {for all } j\in N. \end{array} \end{aligned}$$

Let $f\in {\mathbb {R}} _+^A$ be a maximum flow from the source to the sink with value *v*. A cut can be represented by a two-partition (*S*, *T*) of *N*. The capacity of this cut equals by definition $\sum _{i\in S\cup \{\text {So}\}}\sum _{j\in T\cup \{\text {Si}\}}\text {cap}(i,j)$. This capacity exceeds *x*(*N*), given the reasonability of *x*, as expressed by (3):

$$\begin{aligned} \sum _{i\in S\cup \{\text {So}\}}\sum _{j\in T\cup \{\text {Si}\}}\text {cap}(i,j) = \displaystyle \sum _{i\in S} x_i + \sum _{j\in T}c_{jj} + \sum _{i\in S}\sum _{j\in T}c_{ij} \ge x(S)+x(T)=x(N). \end{aligned}$$

 Ford and Fulkerson (1956) have shown that *v* equals the minimum of all cut capacities. The capacity of the cut $(N,\emptyset )$ equals *x*(*N*), so $v= x(N)$. Define $P\in {\mathbb {R}}_{+}^{N \times N}$ by $p_{ij}=c_{ii}$ if $i=j$ and $p_{ij}=f(i,j)$ if $i\ne j$. It is easy to verify that $P\in {\mathcal {P}} (C)$ with $\alpha ^P(C)=x$. $\square$

Next, we introduce a specific type of transfer schemes.

#### Definition C

Let $C\in \mathcal {L}^N$ and let $\varphi$ be a bankruptcy rule. For all $i\in N$, define $d^i(C)\in {\mathbb {R}}^N$ by

4 $$\begin{aligned} d^i_j(C)={\left\{ \begin{array}{ll} c_{ij} &{}\text { if } j\ne i,\\ 0 &{}\text { if } j=i, \end{array}\right. } \end{aligned}$$

as the vector of claims on agent *i*. Then, $P=(p_{ij})\in {\mathbb {R}}^{N\times N}$ is called a $\varphi$ *-transfer scheme* for *C* if,

(i) for all $$i\in N$$, $$p_{ii}=c_{ii}$$,
(ii) for all $i,j\in N$ with $i\ne j$,
  $$\begin{aligned} p_{ij}=\varphi _j\Big (p_{ii}+\sum _{k\in N\backslash \{i\}}p_{ki},d^i(C)\Big ). \end{aligned}$$

We denote by ${\mathcal {P}}^{\varphi }(C)$ the set of all $\varphi$-transfer schemes.

#### Example 3.3

Let $N=\{1,2,3\}$ and consider the mutual liability problem $C\in {\mathcal {L}}^N$ given by

$$\begin{aligned} C=\begin{bmatrix} 2&\quad 100&\quad 0 \\ 100&\quad 4&\quad 12 \\ 0&\quad 0&\quad 0 \end{bmatrix}. \end{aligned}$$

A $\tau$-transfer scheme is given by

$$\begin{aligned} P=\begin{bmatrix} 2&\quad 8&\quad 0 \\ 6&\quad 4&\quad 6 \\ 0&\quad 0&\quad 0 \end{bmatrix}. \end{aligned}$$

For this, observe e.g. that $p_{23}=\tau _3(p_{22}+p_{12},d^2(C))=\tau _3(12,(100,0,12))=6$. Furthermore, $\alpha ^P=(0,0,6)$. One can check that also the matrix ${\tilde{P}}$ given by

$$\begin{aligned} {\tilde{P}}=\begin{bmatrix} 2&\quad 22&\quad 0 \\ 20&\quad 4&\quad 6 \\ 0&\quad 0&\quad 0 \end{bmatrix} \end{aligned}$$

belongs to ${\mathcal {P}}^{\tau }(C)$. Note that $\alpha ^{{\tilde{P}}}=\alpha ^P$.$\square$

The next lemma shows that a $\varphi$-transfer scheme is indeed a transfer scheme.

#### Lemma 3.4

Let $C\in {\mathcal {L}}^N$ and let $\varphi$ be a bankruptcy rule. Then, ${\mathcal {P}}^{\varphi }(C)\subseteq {\mathcal {P}}(C)$.

#### Proof

Take $P=(p_{ij})\in {\mathcal {P}}^{\varphi }(C)$. It is sufficient to show that condition (ii) in Definition C of a $\varphi$-transfer scheme implies conditions (ii) and (iii) in Definition A of a transfer scheme.

We start with showing (ii). Since $\varphi$ is a general bankruptcy rule, we have that for all $i,j\in N$ with $i\ne j$

$$\begin{aligned} 0&\le \varphi _j\Big (p_{ii}+\sum _{k\in N\backslash \{i\}}p_{ki},d^i(C)\Big )\le d^i_j(C)=c_{ij}, \end{aligned}$$

which implies that

$$\begin{aligned} 0\le p_{ij}\le c_{ij}. \end{aligned}$$

Next we show condition (iii), using the basic properties of a bankruptcy rule. For all $i\in N$,

$$\begin{aligned} \sum _{j\in N\backslash \{i\}}p_{ij}&=\sum _{j\in N\backslash \{i\}}\varphi _j\Big (p_{ii}+\sum _{k\in N\backslash \{i\}}p_{ki},d^i(C)\Big )\\&=\sum _{j\in N}\varphi _j\Big (p_{ii}+\sum _{k\in N\backslash \{i\}}p_{ki},d^i(C)\Big )\\&\le p_{ii}+\sum _{k\in N\backslash \{i\}}p_{ki}. \end{aligned}$$

$$\square $$

A $\varphi$-transfer scheme *P* satisfies an attractive property: in the corresponding $\varphi$-*transfer allocation* $\alpha ^P$ an agent can only receive a positive amount if he paid off all his claimants.

#### Lemma 3.5

Let $P\in {\mathcal {P}}^{\varphi }(C)$ for some $C\in {\mathcal {L}}^N$. Let $i\in N$. If $\alpha _i^{P} > 0$, then

$$\begin{aligned} p_{ij}=c_{ij} \text { for all }j\in N\backslash \{i\}. \end{aligned}$$

#### Proof

Let $\alpha _i^{P}>0$. Then, using Definition B,

$$\begin{aligned} p_{ii}+\sum _{j\in N\backslash \{i\}}(p_{ji}-p_{ij})>0, \end{aligned}$$

i.e.

5 $$\begin{aligned} \sum _{j\in N\backslash \{i\}}p_{ij}<p_{ii}+\sum _{j\in N\backslash \{i\}}p_{ji}. \end{aligned}$$

Moreover, since *P* is a $\varphi$-transfer scheme, for all $j\in N\backslash \{i\}$

$$\begin{aligned} p_{ij}=\varphi _j\left( p_{ii}+\sum _{k\in N\backslash \{i\}}p_{ki},d^i(C)\right) \end{aligned}$$

and consequently

$$\begin{aligned} \sum _{j\in N\backslash \{i\}}p_{ij}=\min \left\{ p_{ii}+\sum _{k\in N\backslash \{i\}}p_{jk},\sum _{j\in N\backslash \{i\}}c_{ij}\right\} . \end{aligned}$$

Using (5) it must be that

$$\begin{aligned} \sum _{j\in N\backslash \{i\}}p_{ij}=\sum _{j\in N\backslash \{i\}}c_{ij} \end{aligned}$$

and using condition (ii) in Definition C of $\varphi$-transfer schemes, it follows that $p_{ij}=c_{ij}$, for all $j\in N\backslash \{i\}$.$\square$

The next theorem shows that one can always find at least one $\varphi$-transfer scheme.

#### Theorem 3.6

Let $C\in {\mathcal {L}}^N$ and let $\varphi$ be a bankruptcy rule. Then, ${\mathcal {P}}^{\varphi }(C)\ne \emptyset$.

#### Proof

Using the following iterative procedure we construct a $\varphi$-transfer scheme for *C*.

For all $i\in N$, set $d^i=d^i(C)$ and set $E^i(0)=c_{ii}$.

Then, recursively define, for all $i\in N$ and $k=1,2,\ldots$,

6 $$\begin{aligned} E^i(k+1)=c_{ii}+\sum _{j\in N\backslash \{i\}}\varphi _i(E^j(k),d^j). \end{aligned}$$

Note that

$$\begin{aligned} E^i(1)=c_{ii}+\sum _{j\in N\backslash \{i\}}\varphi _i(c_{jj},d^j)\ge c_{ii}=E^i(0). \end{aligned}$$

Let $k\ge 1$ and assume that $E^i(k)\ge E^i(k-1)$. Then, by estate monotonicity of $\varphi$ we find that

$$\begin{aligned} E^i(k+1)=c_{ii}+\sum _{j\in N\backslash \{i\}}\varphi _i(E^j(k),d^j)\ge c_{ii}+\sum _{j\in N\backslash \{i\}}\varphi _i(E^j(k-1),d^j)=E^i(k). \end{aligned}$$

Hence, by induction, for all $i\in N$

7 $$\begin{aligned} E^i(0)\le E^i(1)\le E^i(2)\le \ldots \end{aligned}$$

Consider $P=(p_{ij})\in {\mathbb {R}}^{N\times N}$, given by

8 $$\begin{aligned} p_{ij}={\left\{ \begin{array}{ll} c_{ii} &{} \text { for all } i,j\in N \text { with }i=j,\\ \lim \limits _{k\rightarrow \infty }\varphi _j\left( E^i(k),d^i\right) &{} \text { for all } i,j\in N \text { with } i\ne j. \end{array}\right. } \end{aligned}$$

Note that the limit in (8) exists, because $\{E^i(k)\}_{k=0}^{\infty }$ is an increasing sequence that is bounded from above and $\varphi$ is continuous in the estate.

Moreover, condition (ii) in Definition C of a $\varphi$-transfer scheme is satisfied since for all $i,j\in N$ with $i\ne j$, we have that

$$\begin{aligned} p_{ij}=&\lim _{k\rightarrow \infty }\varphi _j\left( E^i(k),d^i\right) \\ =&\varphi _j\left( \lim _{k\rightarrow \infty }E^i(k),d^i\right) \\ =&\varphi _j\left( c_{ii}+\lim _{k\rightarrow \infty }\sum _{\ell \in N\backslash \{i\}}\varphi _i(E^{\ell }(k),d^{\ell }),d^i\right) \\ =&\varphi _j\left( c_{ii}+\sum _{\ell \in N\backslash \{i\}}p_{\ell i},d^i\right) . \end{aligned}$$

The second equality follows from continuity of $\varphi$ in the estate, the third equality follows from (6) and the last equality follows from (8). $\square$

## Hierarchical mutual liability problems

As Example 3.3 shows, in general $\varphi$-transfer schemes need not to be unique. For hierarchical mutual liability problems, however, there is a unique $\varphi$-transfer scheme.

### Theorem 4.1

Let $C\in {\mathcal {L}}^{N,\Delta }$ and let $\varphi$ be a bankruptcy rule. Then, $|{\mathcal {P}}^{\varphi }(C)|=1$.

### Proof

Let $N=\{1,\ldots , n\}$. By the upper triangularity of *C*, we can assume, without loss of generality, that $c_{ij}=0$ if $i>j$. Let $P=(p_{ij})$ and ${\tilde{P}}=({\tilde{p}}_{ij})$ both be $\varphi$-transfer schemes for *C*.

Clearly, if $$i>j$$,

9 $$\begin{aligned} p_{ij}={\tilde{p}}_{ij}=0. \end{aligned}$$

Since

$$\begin{aligned} c_{11}+\sum _{j\in N\backslash \{1\}}p_{j1}=c_{11}+\sum _{j\in N\backslash \{1\}}{\tilde{p}}_{j1}=c_{11}, \end{aligned}$$

the fact that *P* and ${\tilde{P}}$ are $\varphi$-transfer schemes implies for all $j\in N\backslash \{1\}$

$$\begin{aligned} p_{1j}={\tilde{p}}_{1j}=\varphi _j(c_{11},d^1(C)). \end{aligned}$$

Now consider $i\in N$ an assume that for all $g\in \{1,\ldots ,i-1\}$ and $h\in \{1,\ldots ,n\}$,

$$\begin{aligned} p_{gh}={\tilde{p}}_{gh}. \end{aligned}$$

By Eq. (9),

$$\begin{aligned} c_{ii}+\sum _{g\in N\backslash \{i\}}p_{gi}&=c_{ii}+\sum _{g<i}p_{gi}\\&=c_{ii}+\sum _{g<i}{\tilde{p}}_{gi}\\&=c_{ii}+\sum _{g\in N\backslash \{i\}}{\tilde{p}}_{gi} \end{aligned}$$

and thus for all $j\in N\backslash \{i\}$

$$\begin{aligned} p_{ij}&=\varphi _j\left( c_{ii}+\sum _{g\in N\backslash \{i\}}p_{gi},d^i(C)\right) \\&=\varphi _j\left( c_{ii}+\sum _{g\in N\backslash \{i\}}{\tilde{p}}_{gi},d^i(C)\right) ={\tilde{p}}_{ij}. \end{aligned}$$

$$\square $$

This theorem implies that on ${\mathcal {L}}^{N,\Delta }$ a $\varphi$-transfer allocation is uniquely defined for every bankruptcy rule $\varphi$. Hence, we can extend each bankruptcy rule to a hierarchical mutual liability rule.

### Definition D

Let $\varphi$ be a bankruptcy rule. The corresponding *hierarchical* $\varphi$-*based mutual liability rule* $\rho ^{\varphi }:{\mathcal {L}}^{N,\Delta }\rightarrow {\mathbb {R}}^N$ is for all $C\in {\mathcal {L}}^{N,\Delta }$ defined by

$$\begin{aligned} \rho ^{\varphi }(C)=\alpha ^{P}, \end{aligned}$$

where *P* is the unique $\varphi$-transfer scheme for *C*.

An alternative way of using a bankruptcy rule to solve hierarchical mutual liability problems, is the following recursive procedure that we first illustrate by means of an example.

### Example 4.2

Let $N=\{1,\ldots ,4\}$ and consider $C\in {\mathcal {L}}^{N,\Delta }$, given by

$$\begin{aligned} C=\begin{bmatrix} 4&\quad 2&\quad 4&\quad 4\\0&\quad 3&\quad 0&\quad 1\\ 0&\quad 0&\quad 2&\quad 3\\ 0&\quad 0&\quad 0&\quad 2\end{bmatrix}. \end{aligned}$$

In the recursive procedure we start with agent 1, who has no claims on the other agents. His cash, $c_{11}=4$, is divided on the basis of a bankruptcy problem with estate 4 and claims, 2, 4 and 4. Hence we treat this subproblem of the mutual liability problem as a bankruptcy problem (4, (2, 4, 4)). Selecting the Talmud rule $\tau$ as an appropriate bankruptcy rule, we find that $\tau (4,(2,4,4))=(1,1.5,1.5)$. Thus agent 2 gets 1 from agent 1’s cash and agents 3 and 4 both receive 1.5. Correspondingly we can update our (partly) solved mutual liability problem into

$$\begin{aligned} C^{1}=\begin{bmatrix} 4-1-1.5-1.5&0&0&0\\ 0&3+1&0&1\\ 0&0&2+1.5&3\\ 0&0&0&2+1.5\end{bmatrix}=\begin{bmatrix} 0&0&0&0\\ 0&4&0&1\\ 0&0&3.5&3\\ 0&0&0&3.5\end{bmatrix}. \end{aligned}$$

In $C^1$ agent 2 has no claim on agent 1 anymore and we allocate $c^{1}_{22}=4$ on the basis of the bankruptcy problem (4, (0, 1)). Since $\tau (4,(0,1))=(0,1)$, this means that 1 is transferred to agent 4 while agent 2 keeps an amount of 3. Updating leads to

$$\begin{aligned} C^{2}=\begin{bmatrix} 0&\quad 0&\quad 0&\quad 0\\ 0&\quad 3&\quad 0&\quad 0\\ 0&\quad 0&\quad 3.5&\quad 3\\ 0&\quad 0&\quad 0&\quad 4.5\end{bmatrix}. \end{aligned}$$

In the next step an amount of 3 is transferred from 3 to 4, and updating gives

$$\begin{aligned} C^{3}=\begin{bmatrix} 0&\quad 0&\quad 0&\quad 0\\ 0&\quad 3&\quad 0&\quad 0\\ 0&\quad 0&\quad 0.5&\quad 0\\ 0&\quad 0&\quad 0&\quad 7.5\end{bmatrix}. \end{aligned}$$

The diagonal, i.e (0, 3, 0.5, 7.5), of this matrix can be viewed as an allocation which solves this hierarchical mutual liability problem based on a recursive application of the Talmud rule $\tau$.

Importantly, the recursion^2^ implicitly leads to transfer scheme *P* for *C*, where

$$\begin{aligned} P=\begin{bmatrix} 4&1&1.5&1.5\\ 0&3&0&1 \\ 0&0&2&3 \\ 0&0&0&2 \end{bmatrix}. \end{aligned}$$

Note that this is the $\tau$-transfer scheme and that $\alpha ^P=(0,3,0.5,7.5)$. In principle, we could have used a different bankruptcy rule in each step to obtain a transfer scheme.$\square$

The formal definition of how to extend mutual liability rules in the recursive way as described in the previous example is provided below.

### Definition E

Let $C\in {\mathcal {L}}^{N,\Delta }$ and let $\varphi$ be a bankruptcy rule. Set $N=\{1,\ldots ,n\}$ and assume that $c_{ij}=0$ for all $i,j\in N$ with $i>j$. Set $C^0=C$. Recursively, for $j=1,\ldots , n-1$, define $C^{j}\in {\mathcal {L}}^{N,\Delta }$ by

10 $$\begin{aligned} c_{ii}^{j}&={\left\{ \begin{array}{ll} c^{j-1}_{ii} &{}\text { if } i<j\\ c^{j-1}_{ii}+\varphi _{i}\big (c_{jj}^{j-1},(c_{jk})_{k\in \{j+1,\ldots ,n\}}\big ) &{}\text { if } i>j, \end{array}\right. }\nonumber \\ c^{j}_{jj}&=c^{j-1}_{jj}-\sum _{k>j}\varphi _k\big (c^{j-1}_{jj},(c_{jk})_{k\in \{j+1,\ldots ,n\}}\big ),\nonumber \\ c_{ik}^{j}&={\left\{ \begin{array}{ll} 0 &{}\text { if } i=j \text { and } k\ne i,\\ c_{ik}^{j-1} &{}\text { if } i\ne j \text { and } k\ne i.\end{array}\right. } \end{aligned}$$

Finally set

$$\begin{aligned} C^{rec}=C^{n-1}. \end{aligned}$$

Correspondingly, the hierarchical *recursive* $\varphi$-*based mutual liability rule* $\xi ^{\varphi }:{\mathcal {L}}^{N,\Delta }\rightarrow {\mathbb {R}}^N$ is defined by

$$\begin{aligned} \xi ^{\varphi }(C)=\text {diag}(C^{rec}) \end{aligned}$$

for each $C\in {\mathcal {L}}^{N,\Delta }$. Here, diag(*A*) denotes the diagonal of a square matrix *A*.

Interestingly, for every bankruptcy rule $\varphi$, the hierarchical recursive $\varphi$-based mutual liability rule $\xi ^{\varphi }$ and the hierarchical $\varphi$-based mutual liability rule $\rho ^{\varphi }$ coincide.

### Theorem 4.3

For all bankruptcy rules $\varphi$ and all $C\in {\mathcal {L}}^{N,\Delta }$,

$$\begin{aligned} \rho ^{\varphi }(C)=\xi ^{\varphi }(C). \end{aligned}$$

### Proof

Let $C\in {\mathcal {L}}^{N,\Delta }$ and let $\varphi$ be a bankruptcy rule. Set $N=\{1,\ldots ,n\}$ and assume that $c_{ij}=0$ for all $i,j\in N$ with $i>j$. Let $P=(p_{ij})$ be the unique $\varphi$-transfer scheme for *C*. Then, we have that for all $i\in N$

$$\begin{aligned} \rho ^{\varphi }_i(C)=\alpha _i^P&=c_{ii}+ \sum _{j\in N\backslash \{i\}} p_{ji}-\sum _{j\in N\backslash \{i\}}p_{ij}\\&=c_{ii}+\sum _{j=1}^{i-1} p_{ji}-\sum _{j=i+1}^n p_{ij}. \end{aligned}$$

Moreover, for all $i\in N$, $\xi _i^{\varphi }(C)=c^{i}_{ii}$, where $c^i_{ii}$ is determined recursively using (10). Thus it is sufficient to show that for all $i\in N$

11 $$\begin{aligned} c^{i}_{ii}=c_{ii}+\sum _{j=1}^{i-1} p_{ji}-\sum _{j=i+1}^n p_{ij}. \end{aligned}$$

For $i=1$, (11) is satisfied since

$$\begin{aligned} c_{11}^{1}&=c_{11}-\sum _{j=2}^n\varphi _{j}\big (c_{11},(c_{1k})_{k\in \{2,\ldots ,n\}}\big )\\&=c_{11}-\sum _{j=2}^n \varphi _{j}\big (c_{11}+\sum _{k\in N\backslash \{1\}}p_{k1},d^1(C)\big )\\&=c_{11}-\sum _{j=2}^n p_{1j}. \end{aligned}$$

The first equality follows from (10), the second equality holds because $p_{k1}=0$ for all $k\in N\backslash \{1\}$ and the last equality follows from condition (ii) in Definition C of $\varphi$-transfer schemes.

Note that, for all $j\in N\backslash \{1\}$

$$\begin{aligned} c_{jj}^{1}&=c_{jj}+\varphi _{j}\big (c_{11},(c_{1k})_{k\in \{2,\ldots ,n\}}\big )\\&=c_{jj}+p_{1j}. \end{aligned}$$

The proof continues by means of induction. Let $i\le n-1$ and assume that

$$\begin{aligned} c_{jj}^{i-1}={\left\{ \begin{array}{ll}c_{jj}+\sum \limits _{k=1}^{i-1}p_{kj} &{}\text { if } j>i-1\\ c_{jj}+\sum \limits _{k=1}^{j-1}p_{kj}-\sum \limits _{k=j+1}^n p_{jk} &{}\text { if } j\le i-1. \end{array}\right. } \end{aligned}$$

We will prove that

$$\begin{aligned} c_{jj}^i={\left\{ \begin{array}{ll} c_{jj}+\sum \limits _{k=1}^{i}p_{kj} &{}\text { if } j>i\\ c_{jj}+\sum \limits _{k=1}^{i-1}p_{kj}-\sum \limits _{k=j+1}^n p_{jk} &{}\text { if } j=i. \end{array}\right. } \end{aligned}$$

For $j\in \{i+1,\ldots , n\}$, we have that

$$\begin{aligned} c_{jj}^{i}&=c^{i-1}_{jj}+\varphi _{j}\big (c_{ii}^{i-1},(c_{ik})_{k\in \{i+1,\ldots ,n\}}\big )\\&=c_{jj}+\sum _{k=1}^{i-1}p_{kj}+\varphi _{j}\Big (c_{ii}+\sum _{k=1}^{i-1}p_{ki},(c_{ik})_{k\in \{i+1,\ldots ,n\}}\Big )\\&=c_{jj}+\sum _{k=1}^{i-1}p_{kj}+\varphi _{j}\Big (c_{ii}+\sum _{k=1}^{i-1}p_{ki},d^i(C)\Big )\\&=c_{jj}+\sum _{k=1}^{i-1}p_{kj}+p_{ij}\\&=c_{jj}+\sum _{k=1}^{i}p_{kj}, \end{aligned}$$

where the first equality follows from the definition of $\xi$ and the second equality is based on the induction assumption. Similarly one finds

$$\begin{aligned} c^{i}_{ii}&=c^{i-1}_{ii}-\sum _{k=i+1}^n\varphi _k\big (c^{i-1}_{ii},(c_{ik})_{k\in \{i+1,\ldots ,n\}}\big )\\&=c_{ii}+\sum _{k=1}^{i-1}p_{ki}-\sum _{k=i+1}^n\varphi _k\Big (c_{ii}+\sum _{k=1}^{i-1}p_{ki},d^i(C)\Big )\\&=c_{ii}+\sum _{k=1}^{i-1}p_{ki}-\sum _{k=i+1}^n p_{ik}. \end{aligned}$$

$$\square $$

## General mutual liability problems

As seen in Example 3.3, the Talmud rule $\tau$ allows for multiple $\tau$-transfer schemes for a non-hierarchical mutual liability problem. For an arbitrary bankruptcy rule $\varphi$, however, there is always a unique $\varphi$-transfer allocation.

### Theorem 5.1

Let $C\in {\mathcal {L}}^N$, let $\varphi$ be a bankruptcy rule and let $P,{\tilde{P}}\in {\mathcal {P}}^{\varphi }(C)$. Then,

$$\begin{aligned} \alpha ^{P}=\alpha ^{{\tilde{P}}}. \end{aligned}$$

### Proof

On the contrary suppose that $\alpha ^{P}\ne \alpha ^{{\tilde{P}}}$. For notational convenience, set $\alpha ^{P}=\alpha$, $\alpha ^{{\tilde{P}}}={\tilde{\alpha }}$, $E_i = \sum _{j\in N} p_{ji}$ and ${{\tilde{E}}}_i = \sum _{j\in N} {{\tilde{p}}}_{ji}$.

By estate monotonicity, for all $i\in N$: $E_i\le {{\tilde{E}}}_i$ implies that for all $j\in N$ we have $p_{ij}\le {{\tilde{p}}}_{ij}$. As a consequence, for all $i\in N$ we have

12 $$\begin{aligned} p_{ij}<{{\tilde{p}}}_{ij} \text { for some } j\in N \Longrightarrow p_{ij}\le {{\tilde{p}}}_{ij} \text { for all } j\in N. \end{aligned}$$

Let $N=\{1,\ldots ,n\}$. Without loss of generality we assume that, $\alpha _1<{\tilde{\alpha }}_1$. Then ${\tilde{\alpha }}_1>0$, so Lemma 3.5 implies that for all $j\in N$,

13 $$\begin{aligned} {\tilde{p}}_{1j}=c_{1j}. \end{aligned}$$

We will show by induction that for all $i\ge 2$ we have:

14 $$\begin{aligned} \alpha _i=0 \text { and } p_{ij}\le {{\tilde{p}}}_{ij} \text { for all } j\in N. \end{aligned}$$

For this, let $j\in \{2, \ldots ,n\}$ and suppose that (14) is valid for all $i\in \{2, \ldots ,j-1\}$. Note that this is a void assumption in case $j=2$. The agents in $\{1, \ldots ,j-1\}$ together possess less after performing transfer scheme *P* than after performing ${{\tilde{P}}}$, i.e. $\alpha _1$ vs. at least ${{\tilde{\alpha }}} _1$. Hence, there must be agents $k\in \{1, \ldots ,j-1\}$ and $\ell \in \{j, \ldots ,n\}$ such that the net payment from $\ell$ to *k* is greater when ${{\tilde{P}}}$ is applied than when *P* is applied, i.e., such that $p_{\ell k}-p_{k\ell }< {\tilde{p}}_{\ell k}- {\tilde{p}}_{k\ell }.$ Without loss of generality we assume that $\ell =j$. If $k=1$, we have $p_{kj}\le {{\tilde{p}}}_{kj}$ because of (13). If $k>1$, we have $p_{kj}\le {{\tilde{p}}}_{kj}$ by the induction hypothesis for $i=k$. In both cases we find that $p_{jk} < {\tilde{p}}_{jk}$ and hence $\alpha _j =0$ (Lemma 3.5) and $p_{jm}\le {{\tilde{p}}}_{jm}\text { for all }m\in N$ [by (12)]. This proves the induction step and completes the verification of (14).

Equation  (14), however, leads to the contradiction

$$\begin{aligned} \sum _{j\in N}c_{jj} = \sum _{j\in N}\alpha _{j} = \alpha _1<{{\tilde{\alpha }}}_1 \le \sum _{j\in N}{{\tilde{\alpha }}}_{j}= \sum _{j\in N}c_{jj}, \end{aligned}$$

which finishes the proof. $\square$

Theorem 5.1 allows for the following definition.

### Definition F

Let $\varphi$ be a bankruptcy rule. The corresponding $\varphi$-*based mutual liability rule* $\rho ^{\varphi }:{\mathcal {L}}^{N}\rightarrow {\mathbb {R}}^N$ is for all $C\in {\mathcal {L}}^{N}$ defined by

$$\begin{aligned} \rho ^{\varphi }(C)=\alpha ^{P}, \end{aligned}$$

where *P* is a $\varphi$-transfer scheme for *C*.

The final part of this section will provide an axiomatic characterization of $\rho ^{\tau }$ as a $\varphi$-based mutual liability rule on the class ${\mathcal {L}}$ of all mutual liability problems with an arbitrary but finite set of players by extending the concede and divide-principle and consistency for bankruptcy rules to the setting of mutual liability.

In bankruptcy problems the principle of concede and divide is defined for problems with two claimants. However, in a mutual liability problem with two agents, every agent faces only one (possible) claimant. For such mutual liability problems the allocation prescribed by any $\varphi$-based mutual liability rule is unique, as the following lemma states (the proof of this lemma is straightforward and therefore omitted).

### Lemma 5.2

Let ${\mathcal {C}}\in {\mathcal {L}}^N$ with $N=\{1,2\}$. Let $\varphi ^I$ and $\varphi ^{II}$ be bankruptcy rules. Then,

$$\begin{aligned} \rho ^{\varphi ^I}(C)=\rho ^{\varphi ^{II}}(C). \end{aligned}$$

Three entities are involved in a classical bankruptcy situation with two agents, i.e., the bank and the two agents. Since in mutual liability situations agents have multiple roles, it is natural to define a c&d type of axiom in the three agents setting.

### Definition G

A mutual liability rule *f* satisfies the *concede & divide-principle* ($c \& d$) if for each *N* with $|N|=3$ and for each $C\in {\mathcal {L}}^N$, there exists an underlying transfer scheme $P\in {\mathcal {P}}(C)$ such that $f(C)=\alpha ^P$ and for each player $i\in N$, his ‘estate’ $e^i=c_{ii}+\sum _{\ell \ne i}p_{\ell i}$ is allocated among the remaining two players, *j*, *k*, respecting the bankruptcy concede and divide-principle, i.e.

15 $$\begin{aligned} p_{ij}={\left\{ \begin{array}{ll} c_{ij} &{}\text { if } e^i\ge c_{ij}+c_{ik},\\ (e^i-c_{ik})^+ +\frac{e^i-(e^i-c_{ik})^+-(e^i-c_{ij})^+}{2} &{}\text { otherwise. } \end{array}\right. } \end{aligned}$$

### Example 5.3

Reconsider the mutual liability problem $C\in {\mathcal {L}}^N$ of Example 2.1 with $N=\{1,2,3\}$ and *C* given by

$$\begin{aligned} C=\begin{bmatrix} 3&1&4 \\ 2&2&6 \\ 1&0&1 \end{bmatrix}. \end{aligned}$$

Take $P\in {\mathcal {P}}^{\tau }(C)$ given by

$$\begin{aligned} P=\begin{bmatrix} 3&1&4\\ 1&2&2\\ 1&0&1 \end{bmatrix} \end{aligned}$$

with $\rho ^{\tau }(C)=\alpha ^{P}=(0,0,6)$. We check that the entries in *P* satisfy (15). Here, $e^1=p_{11}+p_{21}+p_{31}=5$, $e^2=3$ and $e^3=7$. Both player 1’s and player 3’s estate are sufficient to satisfy their claimants, hence $p_{12}=c_{12}=1$, $p_{13}=4$ and $p_{31}=1$. Player 2’s estate is not sufficient, therefore

$$\begin{aligned} p_{21}=(e^2-c_{23})^+ +\frac{e^2-(e^2-c_{21})^+ -(e^2-c_{23})^+}{2}=0+\frac{3-1-0}{2}=1 \end{aligned}$$

and $$p_{23}=2$$. $$\square $$

Next, we define the property of consistency for a mutual liability rule. This property is defined on the class ${\mathcal {L}}$ of mutual liability problems with arbitrary but finite *N*. The consistency property requires that a reallocation of the total amount which has been allocated to a coalition *T*, on the basis of that rule and an underlying transfer scheme, does not change the initial individual allocations within this coalition.

### Definition H

A mutual liability rule *f* for ${\mathcal {L}}$ is called *consistent* if for all *N* and for all $C\in {\mathcal {L}}^N$ there exists a $P\in {\mathcal {P}}(C)$ such that $f(C)=\alpha ^P$ and such that for all $T\in 2^N\backslash \{\emptyset \}$ with $C^{T,P}\in {\mathcal {L}}^T$,

16 $$\begin{aligned} f(C^{T,P})=f(C)_{|T}, \end{aligned}$$

where $C^{T,P}\in {\mathbb {R}}^{T\times T}$ is defined, for all $i,j\in T$, by

17 $$\begin{aligned} c^{T,P}_{ij}={\left\{ \begin{array}{ll} c_{ij} &{}\text { if } i\ne j,\\ c_{ii}+\sum _{k\in N\backslash T}(p_{ki}-p_{ik}) &{}\text { if } i=j. \end{array}\right. } \end{aligned}$$

Note that there is only the consistency requirement (16) for *T* *if* $C^{T,P}\in {\mathcal {L}}^T$. As is seen in the following example, it can indeed happen that $C^{T,P}\notin {\mathcal {L}}^T$.

### Example 5.4

Let $N=\{1,2,3,4\}$. Reconsider the hierarchical mutual liability problem $C\in {\mathcal {L}}^N$ of Example 2.2, given by

$$\begin{aligned} C=\begin{bmatrix} 4&2&4&4\\0&3&0&1\\ 0&0&2&3\\ 0&0&0&2\end{bmatrix}. \end{aligned}$$

As recursively determined in Example 4.2, the unique $\tau$-transfer scheme *P* for *C* is given by

$$\begin{aligned} P=\begin{bmatrix} 4&1&1.5&1.5 \\ 0&3&0&1 \\ 0&0&2&3\\ 0&0&0&2 \end{bmatrix} \end{aligned}$$

and $$\rho ^{\tau }=(0,3,0.5,7.5)$$.

With $T=\{1,2,4\}$ we have

$$\begin{aligned} C^{T,P}=\begin{bmatrix} 2.5&2&4\\0&3&1\\ 0&0&5 \end{bmatrix}, \end{aligned}$$

which is a mutual liability problem and the unique $\tau$-transfer scheme $P^T$ for $C^{T,P}$ is given by

$$\begin{aligned} P^T=\begin{bmatrix} 2.5&1&1.5 \\ 0&3&1 \\ 0&0&5 \end{bmatrix}, \end{aligned}$$

while $\rho ^{\tau }(C^{T,P})=(0,3,7.5)$. We see that the consistency requirement for this *T* is satisfied. However, with $T=\{1,2,3\}$, we obtain

$$\begin{aligned} C^{T,P}=\begin{bmatrix} 2.5&2&4\\0&2&0\\ 0&0&-1 \end{bmatrix}. \end{aligned}$$

Since $C^{T,P}$ contains negative entries, it is not a mutual liability problem and therefore does not impose a consistency requirement. $\square$

The $\tau$-based mutual liability rule $\rho ^\tau$ satisfies consistency and $c \& d$.

### Theorem 5.5

$\rho ^{\tau }$ is consistent and satisfies $c \& d$ on ${\mathcal {L}}$.

### Proof

We start with proving $c \& d$. Let $C\in {\mathcal {L}}^N$ with $|N|=3$. Let $i\in N$ and set $N\backslash \{i\}=\{j,k\}$. Consider an arbitrary $P\in {\mathcal {P}}^{\tau }(C)$. Obviously $\rho ^{\tau }(C)=\alpha ^P$ by Theorem 5.1. Moreover,

$$\begin{aligned} p_{ij}&=\tau _j(p_{ii}+p_{ji}+p_{ki},d^i(C))\\&=\tau _j(e^i,(c_{ij},c_{ik})). \end{aligned}$$

Since the bankruptcy rule $\tau$ satisfies the $c \& d$ principle for bankruptcy problems, we find that

$$\begin{aligned} p_{ij}={\left\{ \begin{array}{ll} c_{ij} &{}\text { if } e^i\ge c_{ij}+c_{ik},\\ (e^i-c_{ik})^+ +\frac{e^i-(e^i-c_{ik})^+-(e^i-c_{ij})^+}{2} &{}\text { otherwise. } \end{array}\right. } \end{aligned}$$

Next, we show consistency. For this, let $C\in {\mathcal {L}}^N$, consider an arbitrary $P\in {\mathcal {P}}^{\tau }(C)$ and let $T\in 2^N\backslash \{\emptyset \}$ be such that $C^{T,P}\in {\mathcal {L}}^T$. It suffices to show that $\rho ^{\tau }(C)_{|T}=\rho ^{\tau }(C^{T,P})$.

Define $$P^T=(p_{ij}^T)\in {\mathbb {R}}^{T\times T}$$ by

18 $$\begin{aligned} p_{ij}^T={\left\{ \begin{array}{ll} p_{ij} &{}\text { if } i\ne j\\ p_{ii} +\sum _{k\in N\backslash T}(p_{ki}-p_{ik}) &{}\text { if } i=j. \end{array}\right. } \end{aligned}$$

We first show that $P^T\in {\mathcal {P}}^{\tau }(C^{T,P})$, which implies that $\alpha ^{P^T}=\rho ^{\tau }(C^{T,P})$.

For this, note that $c^{T,P}_{ii}=p_{ii}^T$ for all $i\in T$. It remains to prove that for all $i\in T$ and $j\in T\backslash \{i\}$,

$$\begin{aligned} p_{ij}^T=\tau _j\left( p_{ii}^T+\sum _{k\in T\backslash \{i\}}p_{ki}^T,d^i(C^{T,P})\right) . \end{aligned}$$

This is true because for each $i\in T$ and $j\in T\backslash \{i\}$

$$\begin{aligned} p^T_{ij}=p_{ij}&=\tau _j \left( p_{ii}+\sum _{k\in N\backslash \{i\}}p_{ki}, d^i(C)\right) \\&=\tau _j \left( p_{ii}+\sum _{k\in N\backslash \{i\}}p_{ki}-\sum _{k\in N\backslash T}\tau _k\bigg (p_{ii}+\sum _{k\in N\backslash \{i\}}p_{ki},d^i(C)\bigg ), d^i(C)_{|T}\right) \\&=\tau _j \left( p_{ii}+\sum _{k\in N\backslash \{i\}}p_{ki}-\sum _{k\in N\backslash T}p_{ik}, d^i(C)_{|T}\right) \\&=\tau _j\left( p_{ii} +\sum _{k\in N\backslash T}(p_{ki}-p_{ik})+\sum _{k\in T\backslash \{i\}} p_{ki}, d^i(C)_{|T}\right) \\&=\tau _j\left( p^T_{ii}+\sum _{k\in T\backslash \{i\}} p^{T}_{ki}, d^i(C^{T,P})\right) , \end{aligned}$$

where the third equality follows from consistency of the Talmud rule, the fourth equality follows from the fact that $P\in {\mathcal {P}}^{\tau }(C)$, while the last equality follows from (18).

The proof is finished if we show that $\alpha ^{P^T}=\rho ^{\tau }(C)_{|T}$. For this, note that with $i\in T$

$$\begin{aligned} \alpha ^{P^T}_i&=p_{ii}^T+\sum _{j\in T\backslash \{i\}}(p^T_{ji}-p_{ij}^T)\\&=p_{ii}+\sum _{j\in N\backslash T}(p_{ji}-p_{ij})+\sum _{j\in T\backslash \{i\}}(p_{ji}-p_{ij})\\ \phantom {\alpha ^{P^T}_i}&=p_{ii}+\sum _{j\in N\backslash \{i\}}(p_{ji}-p_{ij})\\&=\alpha ^{P}_i=\rho _i^{\tau }(C). \end{aligned}$$

$$\square $$

We conclude this section with a characterization of the $\tau$-based mutual liability rule.

### Theorem 5.6

Let $\varphi$ be a bankruptcy rule. Then, $\rho ^{\varphi }(C)=\rho ^{\tau }(C)$ for all $C\in {\mathcal {L}}$ if and only if $\rho ^{\varphi }$ satisfies consistency and $c \& d$.

### Proof

For the ‘only if’-part, we refer to Theorem 5.5. To prove the ‘if’-part, let $\varphi$ be a bankruptcy rule such that $\rho ^{\varphi }$ satisfies consistency and $c \& d$. As we have seen before, the class ${\mathcal {B}}$ of bankruptcy problems is a subclass of ${\mathcal {L}}$ by identifying each $(E,d)\in {\mathcal {B}}^N$ with $N=\{1,\ldots ,n\}$, with $C(E,d)\in {\mathcal {L}}^{N\cup \{0\},\Delta }$ given byLet *P* be the unique $\varphi$-transfer scheme for *C*(*E*, *d*). Then,

$$\begin{aligned} P= \begin{bmatrix}E&p_{01}&\cdots&p_{0n} \\0&0&\cdots&0\\\vdots&\ddots&\vdots \\0&\cdots&0\end{bmatrix}, \end{aligned}$$

with

$$\begin{aligned} \alpha ^{P}_i={\left\{ \begin{array}{ll}p_{0i} &{} \text { if } i \in N,\\ E-\sum _{j\in N}p_{0j} &{} \text { if } i=0. \end{array}\right. } \end{aligned}$$

Moreover, for all $i\in N$

19 $$\begin{aligned} \rho ^{\varphi }_i(C(E,d))=\alpha ^{P}_i=p_{0i}=\varphi _i(c_{00},d^0(C))=\varphi _i(E,d). \end{aligned}$$

Thus $$\varphi (E,d)=\rho ^{\varphi }(C(E,d))_{|N}$$.

If we can show that

(I) $c \& d$ of $\rho ^{\varphi }$ on ${\mathcal {L}}$ implies $c \& d$ of $\varphi$ on ${\mathcal {B}}$,
(II) consistency of $\rho ^{\varphi }$ on ${\mathcal {L}}$ implies consistency of $\varphi$ on ${\mathcal {B}}$,

then, $\varphi =\tau$ (cf. Aumann and Maschler 1985) and consequently $\rho ^{\varphi }=\rho ^{\tau }$.

For this, we first show that *P* is the unique transfer scheme for *C*(*E*, *d*) that leads to the transfer allocation $\alpha ^P$ and for this reason $c \& d$ and consistency of $\rho ^{\varphi }$ can only have implications on *P*.

Let ${\tilde{P}}=({\tilde{p}}_{ij})\in {\mathcal {P}}(C(E,d))$ be an arbitrary transfer scheme for *C*(*E*, *d*) with ${\tilde{P}}\ne P$. Then,

$$\begin{aligned} {\tilde{P}}= \begin{bmatrix}E&{\tilde{p}}_{01}&\cdots&{\tilde{p}}_{0n} \\0&0&\cdots&0\\\vdots&\ddots&\vdots \\0&\cdots&0\end{bmatrix} \end{aligned}$$

and there must be a player $i\in N$ with ${\tilde{p}}_{0i}\ne p_{0i}$. Hence, $\alpha ^{{\tilde{P}}}\ne \alpha ^{P}$.

With respect to (I), let $N=\{1,2\}$ and $(E,d)\in {\mathcal {B}}^N$. Let $i\in N$ and $\{j\}=N\backslash \{i\}$. We need to show that

$$\begin{aligned} \varphi _i(E,d)={\left\{ \begin{array}{ll} d_i &{} \text { if } E\ge d_{1}+d_{2},\\ (E-d_{j})^+ +\frac{E-(E-d_{i})^+-(E-d_{j})^+}{2} &{}\text { otherwise.} \end{array}\right. } \end{aligned}$$

$C \& d$ on ${\mathcal {L}}$ and (19) imply that, with $c_{0i}=C_{0i}(E,d)$ and $c_{0j}=C_{0j}(E,d)$,

$$\begin{aligned} \varphi _i(E,d){=}\rho ^{\varphi }_i(C(E,d))= & {} {\left\{ \begin{array}{ll} c_{0i} &{} \text { if } e^0{\ge } c_{01}+c_{02},\\ (e^0-c_{0j})^+ +\frac{e^0-(e^0-c_{0i})^+-(e^0-c_{0j})^+}{2} &{}\text { otherwise, } \end{array}\right. }\\= & {} {\left\{ \begin{array}{ll} d_i &{} \text { if } E\ge d_{1}+d_{2},\\ (E-d_{j})^+ +\frac{E-(E-d_{i})^+-(E-d_{j})^+}{2} &{}\text { otherwise.} \end{array}\right. } \end{aligned}$$

With respect to (II), let $(E,d)\in {\mathcal {B}}^N$ and $T\in 2^N\backslash \{\emptyset \}$. We have to prove that

$$\begin{aligned} \varphi (E,d)_{|_T}=\varphi \left( \sum _{j\in T}\varphi _j(E,d),d_{|_T}\right) . \end{aligned}$$

Let $T=\{k_1,\ldots ,k_t\}$. Then, using (17) and (19),Clearly, $C^{T\cup \{0\},P}(E,d)\in {\mathcal {L}}^{T\cup \{0\},\Delta }$ and

$$\begin{aligned} C^{T\cup \{0\},P}(E,d) =C\left( E-\sum _{j\in N\backslash T}\varphi _j(E,d),d_{|T}\right) . \end{aligned}$$

Using consistency, we find for all $i\in T$ that

$$\begin{aligned} \rho ^{\varphi }_i(C(E,d))_{|T\cup \{0\}}=\rho ^{\varphi }_i\left( C\left( E-\sum _{j\in N\backslash T}\varphi _j(E,d),d_{|T}\right) \right) . \end{aligned}$$

By Eq. (19), for all $i\in T$

$$\begin{aligned} {\left\{ \begin{array}{ll} \rho ^{\varphi }_i(C(E,d))&{}=\varphi _i(E,d)\\ \rho ^{\varphi }_i(C(E-\sum _{j\in N\backslash T}\varphi _j(E,d),d_{|T}))&{}=\varphi _i(E-\sum _{j\in N\backslash T}\varphi _j(E,d),d_{|T}) \end{array}\right. } \end{aligned}$$

and therefore,

$$\begin{aligned} \varphi ((E,d))_{|T}=\varphi \left( E-\sum _{j\in N\backslash T}\varphi _j(E,d),d_{|T}\right) =\varphi \left( \sum _{j\in T}\varphi _j(E,d),d_{|T}\right) , \end{aligned}$$

where the last equality follows from (1). $\square$

## On reducing mutual liability problems

In this paper we introduced and analyzed mutual liability problems with nonnegative bilateral claims and nonnegative individual cash levels as an extension of bankruptcy problems. Negative cash levels are assumed to be absent as this could be modeled as the result of a nonnegative claim by an extra party. Clearly, negative claims could be modeled as positive claims by the other party involved. In our view however, the possible reduction of cycles of possible mutual claims is something to be quite careful about. As is illustrated below, this is not an innocent transformation.

The aim of a reduction approach is to transform a general mutual liability problem to a more tractable hierarchical mutual liability problem. The main difference between hierarchical and non-hierarchical mutual liability problems is the (non-)existence of cycles of claims.

In this section we show that, by eliminating these cycles, it is possible to reduce a general mutual liability problem to a hierarchical mutual liability problem, but that such a reduction is not possible without changing the nature of the mutual liability problem. There are choices to be made. Different reduction choices can result in different reduced hierarchical mutual liability problems, as is illustrated in the following example.

### Example 6.1

Let $N=\{1,2,3,4\}$ and let $C\in {\mathcal {L}}^N$ be given by

$$\begin{aligned}C=\begin{bmatrix} 4&5&8&7\\ 1&8&3&12\\ 9&6&6&2\\ 1&1&5&7 \end{bmatrix},\end{aligned}$$

with $$\rho ^{\tau }(C)=(0,2\frac{1}{3},3\frac{1}{3},19\frac{1}{3})$$.

A natural first step in reducing a general mutual liability problem is to assume that on a bilateral level the claims are already settled. Thus for all pairs $i,j\in N$ with $i\ne j$, $c_{ij}c_{ji}=0$. The bilaterally leveled claim matrix $\bar{C}=(\bar{c}_{ij})\in {\mathcal {L}}^N$ is obtained from *C* in the following way

$$\begin{aligned} \bar{c}_{ij}={\left\{ \begin{array}{ll}^+ &{}\text { if } j\ne i\\ c_{ii} &{}\text { if } j=i. \end{array}\right. } \end{aligned}$$

Thus, we eliminate cycles of length 2 and obtain

$$\begin{aligned}\bar{C}=\begin{bmatrix} 4&4&0&6\\ 0&8&0&11\\ 1&3&6&0\\ 0&0&3&7 \end{bmatrix}, \end{aligned}$$

which is still a non-hierarchical mutual liability problem.

Not only can we level claims bilaterally, we can also do this for longer cycles. In the matrix $\bar{C}$ we can find multiple cycles of claims. The longest one, with length 4, goes from player 1 to player 2, then from player 2 to player 4, from player 4 to player 3 and from player 3 back to player 1, see the bold entries in $\bar{C}$ below:

$$\begin{aligned} \bar{C}=\begin{bmatrix} 4&\mathbf {4}&0&6\\ 0&8&0&\mathbf {11}\\ \mathbf {1}&3&6&0\\ 0&0&\mathbf {3}&7 \end{bmatrix}. \end{aligned}$$

Since the lowest claim in this cycle is 1 ($\bar{c}_{31}=1$), we can reduce the cycle by 1, which results in the following non-hierarchical mutual liability problem $C^1$:

$$\begin{aligned} C^1=\begin{bmatrix} 4&3&0&6\\ 0&8&0&\mathbf {10}\\ 0&\mathbf {3}&6&0\\ 0&0&\mathbf {2}&7 \end{bmatrix}. \end{aligned}$$

In $C^1$ we detect another cycle: from 2 to 4, to 3 and back to 2. We can reduce the claims by an amount of 2, with the hierarchical mutual liability problem $C^{1,\Delta }$ as a result. Here,

$$\begin{aligned} C^{1,\Delta }=\begin{bmatrix} 4&3&0&6\\ 0&8&0&8\\ 0&1&6&0\\ 0&0&0&7 \end{bmatrix} \end{aligned}$$

is a hierarchical mutual liability problem; if we rearrange the rows and columns in the order (1, 3, 2, 4), the matrix is upper triangular. We have that $\rho ^{\tau }(C^{1,\Delta })=(0,2.5,5,17.5)\ne \rho ^{\tau }(C)$.

In the mutual liability problem $\bar{C}$, we can also start with another cycle: from 2 to 4, then from 4 to 3 and from 3 back to 2 as shown by the bold entries in $\bar{C}$ below:

$$\begin{aligned} \bar{C}=\begin{bmatrix} 4&4&0&6\\ 0&8&0&\mathbf {11}\\ 1&\mathbf {3}&6&0\\ 0&0&\mathbf {3}&7 \end{bmatrix}. \end{aligned}$$

In this case we can reduce all claims with an amount of 3 and we would immediately end up with the hierarchical mutual liability problem $C^{2,\Delta }$ given by

$$\begin{aligned} C^{2,\Delta }=\begin{bmatrix} 4&4&0&6\\ 0&8&0&8\\ 1&0&6&0\\ 0&0&0&7 \end{bmatrix}. \end{aligned}$$

If we rearrange the players in the order (3, 1, 2, 4), then the matrix is upper triangular. Note that $\rho ^{\tau }(C^{2,\Delta })=(0,2,5,18)$ which is different from both $\rho ^{\tau }(C)$ and from $\rho ^{\tau }(C^{1,\Delta })$. $\square$

In the context of reduction, we would like to conclude with an interesting research question: “Does there exist a liability rule *f* such that for all $C\in {\mathcal {L}}^N$ we have $f(C)=f(C^{bil})$, in which $C^{bil}$ is the reduction of *C* that precisely levels all bilateral claims?”.

## Footnotes

1 Loosely quoted from the framework of the *International Financial Reporting Standards Foundation*.

2 Via the steps $$\begin{bmatrix} 4&\cdot&\cdot&\cdot \\ 0&3&\cdot&\cdot \\ 0&0&2&\cdot \\ 0&0&0&2 \end{bmatrix}\rightarrow \begin{bmatrix} 4&1&1.5&1.5\\ 0&3&\cdot&\cdot \\ 0&0&2&\cdot \\ 0&0&0&2 \end{bmatrix}\rightarrow \begin{bmatrix} 4&1&1.5&1.5\\ 0&3&0&1 \\ 0&0&2&\cdot \\ 0&0&0&2 \end{bmatrix}\rightarrow \begin{bmatrix} 4&1&1.5&1.5\\ 0&3&0&1 \\ 0&0&2&3 \\ 0&0&0&2 \end{bmatrix} $$.
